# Two-Stage Extraction of Clinical Course Information From Psychiatric Discharge Summaries Using Fine-Tuned Large Language Models: Cross-Validation Study

**DOI:** 10.2196/94454

**Published:** 2026-08-06

**Authors:** Chien-Hung Chen, Po-Chang Tseng, Hong-Jie Dai, Chu-Hsien Su, Shi-Heng Wang, Yi-Ling Chien, Wei-Lieh Huang, Chi-Shin Wu, Hsin-Hsi Chen

**Affiliations:** 1 Graduate Institute of Networking and Multimedia, National Taiwan University, Taipei, Taiwan; 2 Institute of Psychiatry, Psychology & Neuroscience, King's College London, London, United Kingdom; 3 Department of Electrical Engineering, College of Electrical Engineering and Computer Science, National Kaohsiung University of Science and Technology, Kaohsiung, Taiwan; 4 National Center for Geriatrics and Welfare Research, National Health Research Institutes, 35, Keyan Road, Zhunan, Miaoli County, 350401, Taiwan, 886 037-206-166 ext 51001; 5 Department of Psychiatry, National Taiwan University Hospital, Taipei, Taiwan; 6 Department of Psychiatry, National Taiwan University Hospital, Yunlin Branch, Douliu, Taiwan; 7 Department of Computer Science and Information Engineering, National Taiwan University, Taipei, Taiwan

**Keywords:** natural language processing, mental disorders, medical record linkage, medical records, time factors

## Abstract

**Background:**

Clinical course information, including disease onset, episode recurrence, and hospitalization history, is essential for psychiatric care and research. However, these data are embedded in unstructured clinical narratives with substantial linguistic variability, making manual extraction labor-intensive and rule-based extraction difficult to scale. Fine-tuned large language models (LLMs) may flexibly extract such information from privacy-sensitive psychiatric records.

**Objective:**

This study aimed to evaluate the performance of LLMs for automatically extracting temporal and clinical course information from psychiatric discharge summaries.

**Methods:**

We analyzed 500 psychiatric discharge summaries from the Integrated Medical Database of National Taiwan University Hospital. A psychiatrist and a natural language processing researcher manually annotated clinical events and temporal information. Four open-source LLMs (LLaMA, MentaLLaMA, OpenBioLLM, and Mistral) were fine-tuned using low-rank adaptation and evaluated using 10-fold cross-validation. A 2-stage framework was developed in which sentence-level extraction of clinical events and temporal information was followed by chart-level prediction of 4 clinical course features: first-episode onset time, episode count, number of psychiatric hospitalizations, and most recent hospitalization. Performance was assessed using precision, recall, *F*_1_-score, accuracy, mean absolute error (MAE), and bootstrap significance testing.

**Results:**

A total of 12,947 sentences were extracted from 500 discharge summaries, yielding 7177 clinical event annotations and 4842 temporal annotations. At the sentence level, Mistral achieved the highest *F*_1_-scores for clinical event extraction, including symptom/episode detection (0.925, 95% CI 0.920‐0.930), hospitalization detection (0.944, 95% CI 0.934‐0.952), and remission/response detection (0.867, 95% CI 0.851‐0.883). Mistral also achieved the highest *F*_1_-scores for most temporal information categories, including age expressions (0.983, 95% CI 0.973‐0.993), relative time expressions (0.953, 95% CI 0.944‐0.963), duration expressions (0.976, 95% CI 0.962‐0.987), and vague temporal expressions (0.888, 95% CI 0.871‐0.905). At the chart level, the proposed 2-stage framework showed the clearest benefit for first-episode onset prediction. Using the 2-stage framework, Mistral achieved the highest point estimate for onset accuracy (0.772), although its performance did not significantly differ from that of LLaMA using the same framework (0.744; bootstrap *P*=.26). For onset prediction, the 2-stage framework significantly outperformed the direct and joint extraction approaches across all evaluated models (all bootstrap *P*≤.002). For other chart-level features, the best-performing approach varied: using the 2-stage framework, Mistral achieved the highest episode count accuracy (0.624; MAE=0.428); using the direct approach, LLaMA achieved the highest hospitalization count accuracy (0.692; MAE=0.379); and using the 2-stage framework, OpenBioLLM achieved the highest most recent hospitalization accuracy (0.868; *F*_1_-score=0.617).

**Conclusions:**

Fine-tuned, locally deployable, open-source LLMs can extract temporal and longitudinal disease course information from psychiatric discharge summaries. The 2-stage framework was most beneficial for first-episode onset prediction and performed competitively across other chart-level features, supporting the use of LLMs to transform heterogeneous psychiatric narratives into structured data for research and decision support.

## Introduction

Understanding the clinical course of psychiatric disorders is essential for effective clinical decision-making and treatment planning [1-3]. Clinical course information includes key temporal and event-based details, such as the onset date, the number of past episodes or relapses, and the frequency of psychiatric hospitalizations. Extracting this information from free-text notes is challenging due to the various ways it is documented (eg, “first psychotic break at 19,” “multiple depressive episodes since 2015,” and *“*no prior psychiatric hospitalizations*”*). Manual retrieval from medical charts is both time-consuming and labor-intensive. Natural language processing (NLP) offers a solution by automating the extraction of these critical details, thereby improving efficiency and reducing human error [4]. Early NLP approaches combined rule-based algorithms with machine learning. For instance, 1 study developed a hybrid NLP system that extracted time expressions and classified relevant text to generate a ranked timeline of probable psychosis onset dates from mental health records [5].

Clinical course information in psychiatric discharge summaries is often expressed in unstructured free text with substantial linguistic variability. For example, the same clinical concept may be documented as “first psychotic break at age 19,” “onset in 2009,” or “symptoms dating back to his early twenties.” Rule-based NLP systems require manually specified patterns for different expression types and may not generalize well to heterogeneous descriptions of psychiatric symptoms, episodes, hospitalizations, and temporal relationships. In addition, although the discharge summaries in this study were predominantly written in English, some records contained Chinese expressions, transliterated terms, or code-mixed content, which further complicated purely rule-based extraction. Large language models (LLMs) are well-suited to this task because they can interpret variable free-text expressions via instruction-following and can be adapted to domain-specific annotation schemas through fine-tuning, as demonstrated by the extraction performance observed in this study. Recent studies demonstrate that LLMs can effectively parse clinical text and answer such questions with reasonable accuracy [6-8]. One study used zero-shot learning with Flan-T5 to extract specifiers, such as severity and remission, for substance use disorders, outperforming traditional rule-based methods in recall by capturing diverse linguistic variations [9].

This study aimed to evaluate locally deployable, open-source LLMs for extracting clinical course information from psychiatric discharge summaries. We developed a 2-stage framework that first extracts sentence-level clinical events and temporal information and then predicts 4 chart-level features: first episode onset time, episode count, number of psychiatric hospitalizations, and most recent hospitalization time. We compared this framework with direct and joint extraction approaches to assess whether explicit sentence-level extraction improves chart-level prediction.

## Methods

### Data Collection

This study used the Integrated Medical Database of National Taiwan University Hospital (NTUH-iMD), a protected electronic medical record system. These data are not publicly available due to patient privacy and ethical restrictions, as stated in the Data Availability Statement. The NTUH-iMD includes electronic medical records such as admission and discharge notes as well as *ICD* (*International Classification of Diseases*) diagnostic codes. This study focused on 4836 discharge summaries from 3087 patients admitted to the psychiatric unit with a principal psychiatric diagnosis (*ICD-9-CM *[*International Classification of Diseases, Ninth Revision, Clinical Modification*]: 290‐319 or *ICD-10-CM* [*International Classification of Diseases, Tenth Revision, Clinical Modification*]: F00-F99) [10]. Discharge summaries in NTUH-iMD are predominantly written in English, consistent with routine clinical documentation practices at NTUH. Although some records contain limited Chinese expressions, transliterated terms, or code-mixed content, the corpus is best characterized as predominantly English clinical text with occasional multilingual elements rather than Chinese-language records. All personal identifying information, including names, addresses, and birthdays, were deidentified, and only sections related to the history of the present illness were included.

Due to the time-consuming nature of manual annotation, 500 discharge notes were selected. As previously described [10], the dataset included 189 (37.8%) notes on schizophrenia, 109 (21.8%) on bipolar disorder, and 117 (23.4%) on unipolar depressive disorder. Four (0.8%) patients had both schizophrenia and unipolar depressive disorder, and 8 (1.6%) had both schizophrenia and bipolar disorder. Additionally, 77 notes covered other diagnoses—dementia, substance use disorder, and anxiety disorders—that were not classified under the main 3 categories [10].

While basic personal information had already been deidentified, we applied an additional deidentification tool [11,12] to replace sensitive details, such as residence, school, workplace, and other elements, that could potentially disclose aspects of the patient’s private life. This tool uses advanced NLP to automatically detect and replace sensitive information [11]. Although effective, its random replacements can affect data completeness and readability. Therefore, after applying the tool, manual review and modification were conducted to ensure both deidentification and the integrity of the data for subsequent analysis.

### Ethical Considerations

This study was approved by the Institutional Review Board of National Taiwan University Hospital (NTUH-201610072RINA). The requirement for informed consent was waived because the study used deidentified retrospective electronic medical records. All data were processed and analyzed in accordance with institutional privacy and data protection regulations.

### Feature Determination

At the sentence level, key clinical course features were identified, including symptoms/episodes indicating disease onset, relapse, recurrence, remission/response, and hospitalization details. Symptoms/episodes encompassed the onset or worsening of psychiatric symptoms and dangerous behaviors such as suicide or violence. Hospitalization included both inpatient treatment and rehabilitation programs. Time information was extracted in formats such as year-month-date, age, vague terms (eg, “childhood” or “this year”), and duration formats (eg, “from A to B,” “between A and B,” or “persisted for A”). These definitions are outlined in Table 1.

**Table 1.** Key clinical course features with examples of clinical events and time formats.

Note segment	Event	Time
Event		
She began to suffer from low mood, insomnia, and suicidal ideation in 2002.	Symptom/episode	Time_YMD: 2002
Under Solian to 600 mg, her symptoms subsided smoothly, with much less olfactory, gustatory, and somatic hallucination, less disturbing behavior, less social withdrawal, and regular daily activity.	Remission/response	—^a^
Therefore, he was admitted to the acute ward in TCPC^b^ in February 2010 for 46 days.	Hospitalization	Time_YMD: 2010‐02; persistence: 46 days
Time		
At the age of 18 years, the patient developed a major depressive episode with mood-congruent psychotic features, precipitated by partner relational problems (the boyfriend had extramarital relations).	Symptom/episode	Age: 18
During the past 2 to 3 weeks, her mood went down again, and she had suicidal ideation (by drug overdose).	Symptom/episode	Ago: 14‐21 days
On March 18, 2007, due to poor drug adherence, her psychotic symptoms aggravated.	Symptom/episode	Time_YMD: 2007-03-18
Besides, dysphoric, labile, easily agitated mood, poor appetite, initial insomnia, and disturbed sleep-wake cycle, loss of energy and interest were also noted in recent months.	Symptom/episode	Vague: recent months
Her depression had been left untreated for 3 to 4 years with gradual but complete improvement.	Remission/response	Persistence: 3‐4 years
So she was admitted again from April 11, 2012, to May 27, 2012.	Hospitalization	Duration: April 11, 2012, to May 27, 2012

a Not available.

b TCPC: Taipei City Psychiatric Center.

At the chart level, we aimed to extract 4 key pieces of information:

1. Onset time: the first appearance of psychiatric symptoms.
2. Episode count: the number of times the patient has experienced psychiatric symptoms, with each episode defined as the period from onset to full or partial remission.
3. Number of psychiatric hospitalizations: the total number of psychiatric hospitalizations includes admissions to acute and chronic wards and rehabilitation centers.
4. Most recent hospitalization time: the time of the most recent psychiatric hospitalization. If no such information is available, it will be recorded as “none.”

Some charts did not provide precise episode counts or hospitalization details (eg, “The patient had several episodes in the past 10 years”). These were excluded from the corresponding analysis.

### Annotation Process

All medical chart annotations were conducted by a board-certified clinical psychiatrist with 20 years of experience (CSW) and a researcher with NLP expertise (CHS). Annotators were instructed to label each sentence based on content, identifying the development or exacerbation of psychiatric symptoms, behaviors related to suicide or violence as “symptom/episode,” treatment response or remission as “response/remission,” admissions to acute psychiatric or rehabilitation wards as “hospitalization,” or to mark the sentence as containing none of the above events. Timing information was also recorded. For example, in the sentence “He had insomnia and poor appetite for one month,” symptoms were annotated along with their duration. Additionally, chart data—including the first onset time, episode count, number of psychiatric hospitalizations, and the date of the most recent hospitalization—were annotated. To assess annotation reliability, a psychiatrist and an NLP researcher independently annotated all training documents. Interrater agreement was evaluated using Cohen κ before consensus adjudication. Disagreements were subsequently resolved through discussion to generate the final reference standard used for model development and evaluation.

Overall interrater agreement was substantial (Cohen κ=0.78). Interrater agreement varied across annotation categories (Table S1 in Multimedia Appendix 1). Agreement was highest for explicit temporal expressions, including calendar dates (κ=0.90), age-based expressions (κ=0.85), and hospitalization events (κ=0.87). In contrast, vague temporal expressions showed substantially lower agreement (κ=0.47), reflecting the inherent ambiguity of phrases such as “during childhood,” “recent years,” or “a long time ago.”

### Model Development

Due to the sensitive nature of psychiatric notes and privacy concerns, we opted for locally deployable models instead of cloud-based LLMs. We used the following models: LLaMA (Llama-3.1-8B-Instruct) [13], MentaLLaMA [14], OpenBioLLM [15], and Mistral (Ministral-8B-Instruct-2410) [16]. We chose these models because they are popular and can be fine-tuned on consumer-level hardware. In addition to the LLMs, we included Bio_ClinicalBERT as a conventional transformer-based baseline for sentence-level information extraction. Bio_ClinicalBERT is a clinical-domain BERT model widely used for clinical named entity recognition tasks [17]. Because Bio_ClinicalBERT is a sequence-labeling model rather than a generative model, it was evaluated only for sentence-level extraction tasks and was not included in chart-level prediction analyses.

**Figure 1.**
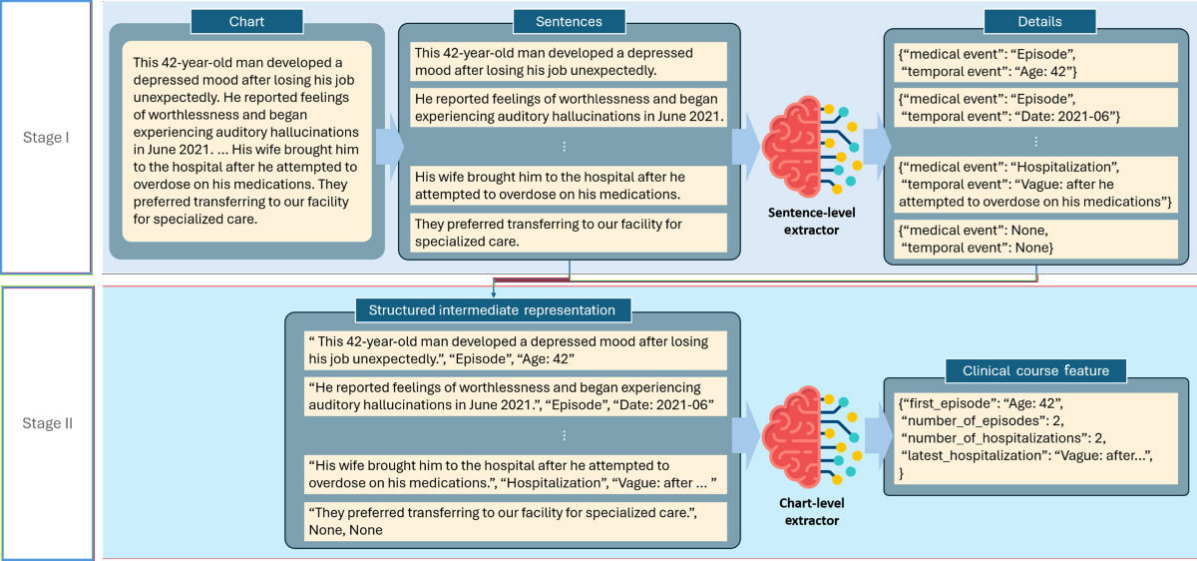
End-to-end pipeline diagram.

Our approach employs a 2-stage extraction method to systematically derive clinical information from psychiatric medical records as illustrated in Figure 1. Stage 1—sentence-level extraction: For each sentence in a discharge note, the sentence-level extractor produces: (1) an event label (“symptom/episode,” “hospitalization,” “remission/response,” or “none”) and (2) associated temporal information (if present), in plain-text format. Stage 2—chart-level aggregation and prediction: all sentence-level predictions are assembled into a structured list maintaining original document order, with the format: [SENTENCE]: <text >| [EVENT]: <label >| [TIME]: <temporal info or “none”>. This structured list is provided as input to the Stage 2 chart—level extractor, which synthesizes this information to produce the 4 clinical course features (onset time, episode count, hospitalization count, and last hospitalization time).

**Figure 2.**
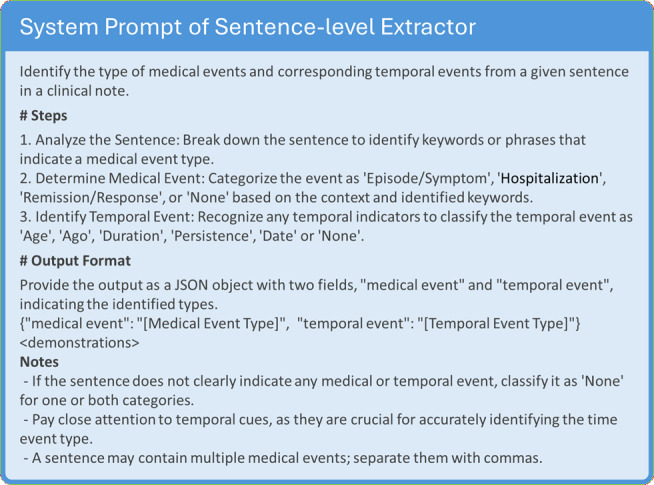
System prompt for the sentence-level extractor.

For sentence-level extraction, we develop a specialized system prompt that clearly communicates the task objective: accurately classifying medical and temporal event types from sentences in clinical notes, as shown in Figure 2. The design of this prompt focuses on guiding the model to reliably identify and categorize relevant medical events, including episodes, symptoms, hospitalizations, and temporal indicators. By emphasizing context and temporal cues, the prompt facilitates precise extraction, ensuring consistency across varying clinical scenarios.

**Figure 3.**
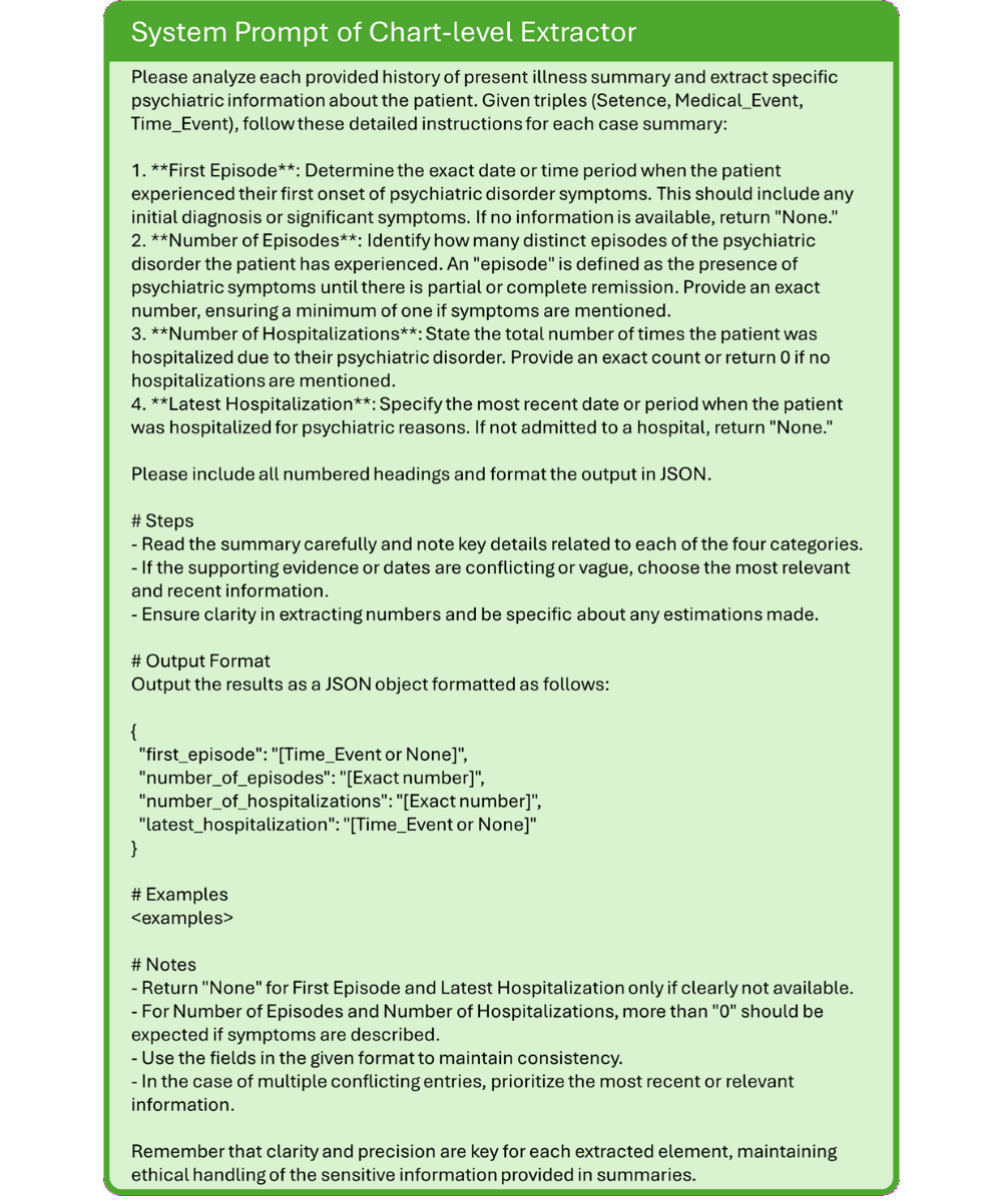
System prompt for the chart-level extractor.

In the second stage, the chart-level extractor synthesizes aggregated sentence-level predictions to derive detailed psychiatric summaries from patient records. The chart-level prompt was crafted to systematically guide the extraction of key clinical features, including the first occurrence of symptoms, the number of episodes, hospitalization count, and the timing of the most recent hospitalization. This design aims to standardize the summarization process, clarify the criteria for data extraction, and minimize ambiguity, thereby enabling robust, consistent results suitable for clinical analysis. The detailed system prompt is shown in Figure 3.

Both extractors use low-rank adaptation (LoRA) [18] for fine-tuning. Annotated data were explicitly structured for fine-tuning, converting labeled annotations into corresponding prompt-response pairs aligned with each designed prompt. This structured data approach facilitates supervised learning and improves model performance.

The fine-tuning configurations were optimized to ensure training efficiency and accuracy. The batch size was set to 1, with gradient accumulation across 16 steps. We set the learning rate to 0.0001 and trained the model for 5 epochs, using a warm-up ratio of 0.1. The maximum sequence length was set to 8192 tokens. For LoRA, we set the rank to 8, the α to 16, and applied a dropout rate of 0.05. The LoRA hyperparameters were selected via a grid search over rank ∈ {4, 8, 16}, alpha ∈ {8, 16, 32}, and dropout ∈ {0.01, 0.05, 0.1}, with validation performance averaged across the first 3 folds.

All experiments were conducted on a single NVIDIA A6000 48 GB GPU. Fine-tuning each model for 1 fold required approximately 2 to 3 hours (sentence-level extractor: ~1.5 h; chart-level extractor: ~1 h). Inference (prediction) took approximately 20 minutes per fold per model. Models can run on consumer hardware with ≥24 GB of VRAM using 4-bit quantization, though fine-tuning would require proportionally more time.

### Evaluation Metrics and Statistical Comparison

Sentence-level extraction performance was evaluated separately for clinical events and temporal information. Precision, recall, and *F*_1_-score were calculated for each annotation category. Performance was estimated using 10-fold cross-validation, and the mean performance across folds was reported. Clinical event extraction was evaluated for symptom/episode, hospitalization, and remission/response events. Temporal extraction was evaluated for calendar dates, age expressions, relative time expressions, duration, persistence, and vague temporal expressions.

Chart-level performance was evaluated for four clinical course features: first-episode onset time, number of episodes, number of hospitalizations, and most recent hospitalization. For temporal outcomes (first-episode onset time and most recent hospitalization), performance was assessed using exact-match accuracy and macroaveraged *F*_1_-score. Predicted temporal expressions were normalized prior to evaluation, and partial matches (eg, “2010” vs “2010‐03”) were considered incorrect. For count-based outcomes (number of episodes and number of hospitalizations), both exact-match accuracy and mean absolute error (MAE) were reported. Lower MAE values indicate better performance.

To assess whether performance differences between methods were statistically meaningful, pairwise comparisons were conducted using bootstrap resampling. For each comparison, 1000 bootstrap samples were generated by resampling discharge summaries with replacement from the evaluation set, and 2-sided bootstrap *P* values were calculated from the resulting distribution of performance differences. Comparisons focused on the proposed 2-stage framework vs the direct and Joint approaches.

### Additional Analyses

#### Oracle Analysis

To quantify the contribution of sentence-level extraction errors to overall chart-level performance, an oracle analysis was performed. In this analysis, gold-standard sentence-level annotations were provided directly to the chart-level extraction stage, replacing model-generated sentence predictions. The resulting performance represents an approximate upper bound of the second-stage chart-level extractor under perfect sentence-level extraction.

#### Prompt Sensitivity Analysis

A prompt sensitivity analysis was conducted to evaluate the effects of prompt design choices. Four prompt variants were examined: (1) the initial structured prompt, (2) a version without few-shot examples, (3) a version incorporating chain-of-thought reasoning instructions, and (4) the final simplified plain-text output format used in the proposed framework. Sentence-level *F*_1_-scores were compared across prompt variants.

## Results

### Data Collection

In an analysis of 500 discharge notes, a total of 12,947 sentences were extracted, with sentence counts ranging from 4 to 112 per note, averaging 25.9 (SD 17.3) sentences. Of these, 7177 sentences contained clinical events, and 4842 included time-related information.

### Sentence-Level Information Extraction Results

Fine-tuning substantially improved sentence-level extraction performance across all evaluated LLMs (Table S2 in Multimedia Appendix 1). While performance gains varied across models, LoRA fine-tuning consistently increased *F*_1_-scores for symptom/episode, hospitalization, and remission/response extraction. The largest improvements were observed in OpenBioLLM and MentaLLaMA, which demonstrated limited zero-shot performance but achieved competitive results after fine-tuning. These findings underscore the importance of task-specific adaptation when applying LLMs to psychiatric clinical narratives.

Tables 2 and 3 present the sentence-level extraction performance of all models after fine-tuning. Mistral achieved the highest *F*_1_-scores across most clinical event categories, including symptom/episode (0.925), hospitalization (0.944), and remission/response (0.867). It also demonstrated the strongest performance for most temporal information categories, including age (0.983), relative time expressions (0.953), duration (0.976), time references (0.991), and vague temporal expressions (0.888). Bio_ClinicalBERT, a conventional transformer-based sequence-labeling model, achieved competitive performance for several extraction tasks but was generally outperformed by the best-performing fine-tuned LLMs. Nevertheless, LLaMA and OpenBioLLM showed performance comparable to Mistral for duration and time references. Persistent and vague temporal expressions remained the most challenging categories across all models, although Mistral achieved the highest performance in both tasks.

**Table 2.** Sentence-level performance for clinical event extraction across models^a^.

Model	Symptom/episode			Hospitalization			Remission/response		
	Precision	Recall	*F*_1_-score (95% CI)	Precision	Recall	*F*_1_-score (95% CI)	Precision	Recall	*F*_1_-score (95% CI)
LLaMA	0.875	0.885	0.880 (0.874‐0.886)	0.945	0.896	0.920 (0.908‐0.931)	0.795	0.786	0.790 (0.771‐0.808)
MentaLLaMA	0.875	0.875	0.875 (0.869‐0.882)	0.902	0.909	0.905 (0.894‐0.916)	0.784	0.763	0.773 (0.753‐0.794)
OpenBioLLM	0.877	0.867	0.872 (0.865‐0.878)	0.927	0.913	0.920 (0.909‐0.930)	0.808	0.753	0.779 (0.759‐0.799)
Mistral	0.923	0.926	0.925 (0.920‐0.930)^b^	0.982	0.908	0.944 (0.934‐0.952)^b^	0.880	0.854	0.867 (0.851‐0.883)^b^
Bio_ClinicalBERT	0.865	0.864	0.864 (0.858‐0.871)	0.906	0.915	0.910 (0.898‐0.921)	0.787	0.725	0.755 (0.734‐0.775)

a Higher values indicate better performance. All large language models were fine-tuned using low-rank adaptation and evaluated using the same 10-fold cross-validation framework.

b Indicates the highest *F*_1_-score for each event category.

**Table 3.** Sentence-level performance for temporal information extraction across models^a^.

Model	Age *F*_1_ (95% CI)	Ago *F*_1_ (95% CI)	Duration *F*_1_ (95% CI)	Persistence *F*_1_ (95% CI)	Time *F*_1_ (95% CI)	Vague *F*_1_ (95% CI)
LLaMA	0.961 (0.946‐0.976)	0.926 (0.915‐0.939)	0.963 (0.947‐0.977)	0.529 (0.448‐0.596)	0.988 (0.985‐0.991)	0.815 (0.791‐0.837)
MentaLLaMA	0.954 (0.938‐0.969)	0.925 (0.913‐0.938)	0.961 (0.945‐0.975)	0.571 (0.493‐0.641)	0.988 (0.985‐0.991)	0.812 (0.788‐0.835)
OpenBioLLM	0.960 (0.945‐0.973)	0.927 (0.914‐0.938)	0.966 (0.951‐0.981)	0.513 (0.431‐0.591)	0.989 (0.985‐0.992)	0.808 (0.783‐0.829)
Mistral^b^	0.983 (0.973‐0.993)	0.953 (0.944‐0.963)	0.976 (0.962‐0.987)	0.740 (0.684‐0.797)	0.991 (0.988‐0.993)	0.888 (0.871‐0.905)
Bio_ClinicalBERT	0.950 (0.932‐0.965)	0.924 (0.911‐0.936)	0.969 (0.953‐0.982)	0.605 (0.532‐0.665)	0.986 (0.983‐0.989)	0.797 (0.772‐0.820)

a Values are *F*_1_-scores. Higher values indicate better performance. All LLMs were fine-tuned using LoRA and evaluated under the same 10-fold cross-validation framework.

b The values of Mistral model have the highest *F*_1_-score for each temporal category.

### Clinical Course Information Extraction Results

Table 4 summarizes chart-level performance in predicting key clinical course features, whereas Table 5 presents bootstrap-based pairwise comparisons between the proposed 2-stage framework and alternative approaches. Across all models, the 2-stage framework achieved the highest performance for the first-episode onset prediction. Mistral_Ours achieved the best onset accuracy (0.772), followed by LLaMA_Ours (0.744), OpenBioLLM_Ours (0.738), and MentaLLaMA_Ours (0.734). Detailed precision and recall results for the proposed 2-stage framework (ours) are provided in Table S3 in Multimedia Appendix 1.

For all 4 models, the 2-stage framework significantly outperformed both the direct and joint approaches for onset prediction (all bootstrap *P*≤.002). For episode count prediction, Mistral_Ours (accuracy=0.624) and LLaMA_Ours (accuracy=0.618) achieved comparable performance, with no statistically significant difference between them (bootstrap *P*=.85). For hospitalization count prediction, improvements over the direct approach were limited, with no significant differences observed for either LLaMA (*P*=.56) or Mistral (*P*=.99). However, the 2-stage framework consistently outperformed the joint approach for hospitalization count prediction across all evaluated models. For the most recent hospitalization, the 2-stage framework significantly outperformed the direct approach for all models (all bootstrap *P*<.05), although differences between the 2-stage and joint approaches were generally smaller.

**Table 4.** Chart-level performance for clinical course feature extraction using direct, joint, and 2-stage approaches^a^.

Method	Onset accuracy	Onset *F*_1_-score	Episode count accuracy	Episode count MAE^b^	Hospitalization count accuracy	Hospitalization count MAE	Latest hospitalization accuracy	Latest hospitalization *F*_1_-score
LLaMA_Direct^c^	0.63	0.368	0.542	0.532	0.692^d^	0.379^d^	0.754	0.405
LLaMA_Joint^e^	0.64	0.426	0.522	0.536	0.584	0.516	0.832	0.538
LLaMA_Ours^f^	0.744	0.547	0.618	0.475	0.67	0.452	0.866	0.628^d^
MentaLLaMA_Direct	0.558	0.308	0.484	0.653	0.568	0.627	0.742	0.385
MentaLLaMA_Joint	0.484	0.318	0.306	0.831	0.404	0.805	0.734	0.377
MentaLLaMA_Ours	0.734	0.531	0.504	0.598	0.61	0.55	0.858	0.591
OpenBioLLM_Direct	0.596	0.356	0.532	0.578	0.644	0.473	0.788	0.459
OpenBioLLM_Joint	0.644	0.418	0.488	0.588	0.612	0.454	0.838	0.547
OpenBioLLM_Ours	0.738	0.538	0.574	0.483	0.682	0.428	0.868^d^	0.617
Mistral_Direct	0.648	0.388	0.58	0.474	0.674	0.427	0.784	0.436
Mistral_Joint	0.614	0.379	0.522	0.519	0.606	0.388	0.83	0.538
Mistral_Ours	0.772^d^	0.582^d^	0.624^d^	0.428^d^	0.676	0.436	0.858	0.605

a Onset indicates the first-episode onset time and last hospitalization denotes the most recent psychiatric hospitalization. Accuracy and *F*_1_-score were used for temporal prediction tasks, whereas both exact-match accuracy and mean absolute error (MAE) were reported for count-based tasks. Higher values indicate better performance for accuracy and *F*_1_-score; lower values indicate better performance for MAE.

b MAE: mean absolute error.

c Direct: direct chart-level prediction.

d indicates the best performance for each temporal category.

e Joint: single-stage joint extraction.

f Ours: proposed 2-stage framework.

**Table 5.** Bootstrap-based pairwise comparisons of chart-level accuracy^a^.

Comparison	Mean (ours)	Mean (baseline)	Δ	*P *(bootstrap)
Onset time				
LLaMA_Ours^b^ vs Direct^c^	0.744	0.630	+0.114	<.001
LLaMA_Ours vs Joint^d^	0.744	0.640	+0.104	.001
Mistral_Ours vs Direct	0.772	0.648	+0.124	<.001
Mistral_Ours vs Joint	0.772	0.614	+0.158	<.001
OpenBioLLM_Ours vs Direct	0.738	0.596	+0.142	<.001
OpenBioLLM_Ours vs Joint	0.738	0.644	+0.094	.002
MentaLLaMA_Ours vs Direct	0.734	0.558	+0.176	<.001
MentaLLaMA_Ours vs Joint	0.734	0.484	+0.250	<.001
Mistral_Ours vs LLaMA_Ours	0.772	0.744	+0.028	.26
Episode count				
LLaMA_Ours vs Direct	0.618	0.542	+0.076	.03
LLaMA_Ours vs Joint	0.618	0.522	+0.096	<.001
Mistral_Ours vs Direct	0.624	0.580	+0.044	.27
Mistral_Ours vs Joint	0.624	0.522	+0.102	.03
OpenBioLLM_Ours vs Joint	0.574	0.488	+0.086	.006
MentaLLaMA_Ours vs Joint	0.504	0.306	+0.198	<.001
Mistral_Ours vs LLaMA_Ours	0.624	0.618	+0.006	.85
Hospitalization count				
LLaMA_Ours vs Direct	0.670	0.692	−0.022	.56
LLaMA_Ours vs Joint	0.670	0.584	+0.086	.02
Mistral_Ours vs Direct	0.676	0.674	+0.002	.99
Mistral_Ours vs Joint	0.676	0.606	+0.070	.02
OpenBioLLM_Ours vs Joint	0.682	0.612	+0.070	.046
MentaLLaMA_Ours vs Joint	0.610	0.404	+0.206	<.001
Mistral_Ours vs LLaMA_Ours	0.676	0.670	+0.006	.91
Last hospitalization				
LLaMA_Ours vs Direct	0.866	0.754	+0.112	.001
LLaMA_Ours vs Joint	0.866	0.832	+0.034	.28
Mistral_Ours vs Direct	0.858	0.784	+0.074	.03
Mistral_Ours vs Joint	0.858	0.830	+0.028	.44
OpenBioLLM_Ours vs Direct	0.868	0.788	+0.080	.006
OpenBioLLM_Ours vs Joint	0.868	0.838	+0.030	.31
MentaLLaMA_Ours vs Direct	0.858	0.742	+0.116	.006
MentaLLaMA_Ours vs Joint	0.858	0.734	+0.124	.004
Mistral_Ours vs LLaMA_Ours	0.858	0.866	−0.008	.86

a Values represent chart-level accuracy averaged across 10-fold cross-validation. Δ denotes the difference in accuracy between the proposed 2-stage framework (ours) and the comparison method. Statistical significance was assessed using 1000 bootstrap resamples. Lower *P* values indicate stronger evidence that the observed accuracy difference was not due to random variation.

b Ours: proposed 2-stage framework.

c Direct: direct chart-level prediction.

d Joint: single-stage joint extraction.

### Results of Additional Analyses

#### Oracle Analysis Results

Oracle analysis revealed heterogeneous effects across models (Table S4 in Multimedia Appendix 1). For onset time prediction, oracle inputs modestly improved performance for OpenBioLLM (0.776 vs 0.738) and MentaLLaMA (0.762 vs 0.734), indicating that sentence-level extraction errors contributed to downstream performance limitations in these models. In contrast, oracle performance was comparable to or lower than that of the complete pipeline for LLaMA (0.728 vs 0.744) and Mistral (0.702 vs 0.772). For episode count and hospitalization count, oracle and chain performances were highly similar across all models, suggesting that these tasks were relatively robust to sentence-level extraction errors. For the most recent hospitalization, oracle performance was consistently lower than that of the complete pipeline (eg, Mistral: 0.796 vs 0.858; LLaMA: 0.846 vs 0.866).

#### Prompt Sensitivity Analysis Results

Prompt design had a measurable impact on extraction performance (Table S5 in Multimedia Appendix 1). The initial structured prompt produced the lowest overall performance, whereas the simplified plain-text format adopted in the final system achieved the best overall results across most clinical event and temporal extraction tasks. Removing few-shot examples improved performance for several common extraction categories, although performance for persistence and vague temporal expressions declined. While chain-of-thought prompting improved performance on some temporal extraction tasks, it did not consistently improve overall performance. These findings suggest that prompt structure and output format can substantially influence extraction quality in prompt-only evaluations and informed the final prompt design used for subsequent fine-tuning.

## Discussion

### Principal Results

As demonstrated in this study, modern LLMs provide a significant step forward in improving the efficiency and accuracy of the analysis of the course of psychiatric disorder. Our results highlight the potential of LLMs to extract critical clinical information from electronic health records, significantly reducing the manual burden on clinicians while enhancing the quality of the extracted data.

A notable finding of this study was the consistent advantage of the proposed 2-stage framework over both direct and joint extraction approaches. One possible explanation is that psychiatric discharge summaries often contain multiple temporally distributed events, diagnoses, remissions, and hospitalizations within a single document. Direct prediction requires the model to simultaneously identify relevant events, resolve temporal information, and infer chart-level clinical course features from the entire note, creating a highly complex reasoning task. Although the joint approach explicitly incorporates sentence-level extraction, it still requires event extraction and chart-level prediction to be performed within a single prompt-response process. In contrast, the proposed framework decomposes the task into 2 sequential steps. By first converting free-text narratives into structured event-time representations and then performing chart-level reasoning on the structured outputs, the model can focus on a simpler objective at each stage. This decomposition likely reduces cognitive load, improves interpretability, and facilitates the aggregation of longitudinal clinical information, particularly for first-episode onset prediction, where the largest performance gains were observed. The greatest improvements were observed for first-episode onset prediction, suggesting that separating temporal information extraction from chart-level reasoning is particularly beneficial for reconstructing longitudinal disease trajectories.

However, oracle analyses suggested that chart-level performance was not determined solely by sentence-level extraction accuracy. Although replacing model-generated sentence-level outputs with gold-standard annotations improved performance for some models and tasks, the improvement was not consistent. This suggests that many chart-level errors arose from the second-stage aggregation and reasoning process, such as selecting the correct onset time, distinguishing episodes from hospitalizations, and integrating multiple temporally distributed events across a discharge summary. In addition, the chart-level extractor was developed using model-generated sentence-level outputs, and gold-standard annotations may differ in format or information density from these predicted inputs. These findings suggest that future improvements should target both sentence-level extraction and chart-level clinical reasoning.

### Comparison With Prior Work

Our study did not use cloud-based APIs, such as OpenAI’s GPT, due to the highly sensitive nature of psychiatric notes, in which data privacy is a critical concern. As a result, we were unable to compare the performance of OpenAI’s GPT with other LLMs. Instead, we selected LLaMA and Mistral for this study, as they can be deployed locally and fine-tuned on private data, mitigating privacy risks. While LLaMA and similar models offer greater control and customization, they often require fine-tuning or retrieval augmentation to achieve performance comparable to larger proprietary models such as GPT-4 [19]. To address this, researchers have developed domain-specific LLMs for mental health, such as MentaLlama, which integrate psychiatric knowledge through fine-tuning [14]. With proper adaptation, open models can significantly reduce the performance gap.

To further investigate the impact of domain-specific open models, we evaluated the performance of MentaLlama [14] and OpenBioLLM [15]. However, the results did not show a clear improvement over LLaMA 3.1. A possible explanation is that MentaLlama is based on LLaMA 2, while OpenBioLLM is built on LLaMA 3; their effectiveness may, therefore, be limited by the capabilities of their respective base models.

Recent studies highlight the potential of Mistral-7B in clinical text extraction. A fine-tuned version of the model achieved 91% accuracy in identifying key clinical history details, closely matching GPT-4’s 92%, with no statistically significant difference [20]. Despite being an order of magnitude smaller than GPT-4, Mistral-7B, optimized with prompt engineering and in-context learning, reached near-human agreement (κ≈0.75) with radiologists to extract structured history elements.

These findings suggest that while GPT-4 excels in complex reasoning tasks, smaller open models such as LLaMA and Mistral can be competitive when fine-tuned for domain-specific applications. The choice of model depends on balancing performance, resource availability, and data security. In contrast, LLaMA and Mistral provide greater control through local deployment and fine-tuning, albeit with some trade-offs in raw performance.

### Error Analysis

Error analysis revealed several recurring sources of chart-level extraction errors. The most common error category was ambiguous temporal information (55/179, 30.7%), followed by missing information in the source notes (27/183, 14.8%), episode-hospitalization conflation (19/166, 11.4%), and confusion arising from multiple diagnoses with distinct onset times (11/108, 10.2%). Errors related to multiple clinical events described within a single sentence accounted for an additional 8.0% (9/112).

Missing information in the source notes often limited the accuracy of extraction, regardless of the model’s capabilities. For example, in the note “Since then, the patient has been repeatedly admitted to psychiatric services due to unstable mood and suicide attempts,” the number of hospitalizations was not specified, preventing accurate extraction. Ambiguous temporal information was another major source of error. In one case, a patient was evaluated in the emergency department and subsequently admitted, but no explicit hospitalization date was documented. The model occasionally inferred the outpatient visit date as the admission date, despite the absence of a clearly stated admission date.

The complexity of psychiatric narratives also contributed to extraction errors. When multiple diagnoses with distinct onset times were described in the same note, the model sometimes failed to correctly associate symptom onset with the appropriate diagnosis. For example, in a note describing autism diagnosed at the age of 4 years and mood symptoms beginning at the age of 15 years, the model occasionally conflated the onset times of the 2 conditions. Similarly, when multiple clinical events were described within a single sentence, the model sometimes captured only one event while overlooking others. For instance, in the sentence “Psychotic symptoms improved in March 2010 but worsened in May 2010,” the model often identified the symptom exacerbation but failed to recognize the preceding improvement.

Finally, episode-hospitalization conflation represented a distinct error pattern. In notes describing multiple psychiatric episodes alongside several hospital admissions, the model occasionally interpreted each episode as a separate hospitalization, leading to the overestimation of hospitalization counts. Overall, these findings suggest that many chart-level errors arise from incomplete documentation and the inherent complexity of psychiatric narratives rather than simple failures of event recognition.

### Limitations

Despite the promising results, several limitations were identified in this study. First, the dataset used was limited to discharge notes from a single hospital, which may not fully represent the diversity of psychiatric documentation across different clinical settings. Further research is needed to validate the model’s performance on larger and more diverse datasets, including outpatient and emergency room records, to ensure broader applicability. Second, the dataset is best characterized as primarily English clinical text with occasional code-mixed elements rather than fully Chinese-language records. Because the evaluated models were primarily pretrained on English corpora and the study used predominantly English discharge summaries, the generalizability of our findings to predominantly Chinese-language psychiatric records or other multilingual clinical settings remains uncertain. Future studies should evaluate the proposed framework on datasets with varying language compositions and across different health care settings. Third, the study does not differentiate between onset times for multiple diagnoses when patients have comorbid psychiatric conditions. This omission can lead to inaccuracies in real-world applications. Fourth, we identified only 4 items related to the clinical course. Expanding the scope of extracted features to include additional relevant clinical indicators—such as treatment responses, adverse effects for each regimen, drug compliance assessments, and identification of precipitating factors for relapse and recurrence—would provide a more comprehensive view of the patient’s psychiatric trajectory. Finally, several risks associated with deploying LLMs in clinical settings warrant explicit consideration. LLMs can generate plausible but incorrect outputs, and the extracted information should therefore be treated as decision-support rather than authoritative clinical data. We emphasize that the present system is a research prototype and must undergo prospective clinical validation before any real-world deployment. All predictions should be reviewed by qualified clinicians before informing treatment decisions.

### Conclusions

This study demonstrates the potential of LLMs to automate the extraction of key clinical features from psychiatric discharge summaries. The model effectively captures structured data such as hospitalization events and onset dates, though further refinement is needed to handle vague temporal information and complex clinical nuances. Despite these challenges, LLMs show promise in enhancing psychiatric care by equipping clinicians with efficient, accurate tools for managing psychiatric disorders. As LLM technology advances, its integration into routine practice may improve the quality of care and patient outcomes.

## Supplementary material

10.2196/94454 Multimedia Appendix 1 Supplementary tables supporting the main analyses.

## Abbreviations

*ICD*: *International Classification of Diseases*
*ICD-10-CM*: *International Classification of Diseases, Tenth Revision, Clinical Modification*
*ICD-9-CM*: *International Classification of Diseases, Ninth Revision, Clinical Modification*
LLM: large language model
LoRA: low-rank adaptation
MAE: mean absolute error
NLP: natural language processing
NTUH-iMD: Integrated Medical Database of National Taiwan University Hospital
