# Supplementary material for: Two-Stage Extraction of Clinical Course Information From Psychiatric Discharge Summaries Using Fine-Tuned Large Language Models: Cross-Validation Study
Source: JMIR Form Res. 2026 Aug 6;10:e94454. doi: 10.2196/94454 (PMC13446380; doi:10.2196/94454)
Supplement: Supplementary file 1 [file formative-v10-e94454-s001.docx]

### Supplementary Material

### Supplementary Table S1. Inter-Rater Agreement for Temporal Information and Clinical Event Annotations

| Annotation Category | Final Positive Cases After Consensus (n) | Both Positive | Annotator A Only | Annotator B Only | Both Negative | Cohen's κ |
| --- | --- | --- | --- | --- | --- | --- |
| Temporal Information |  |  |  |  |  |  |
| Calendar date | 2,494 | 2,094 | 186 | 197 | 10,470 | 0.90 |
| Age | 359 | 338 | 59 | 52 | 12,498 | 0.85 |
| Relative time ("ago") | 902 | 774 | 169 | 143 | 11,862 | 0.82 |
| Duration | 312 | 221 | 123 | 94 | 12,509 | 0.66 |
| Persistence | 148 | 110 | 81 | 56 | 12,700 | 0.61 |
| Vague temporal expression | 627 | 279 | 293 | 280 | 12,095 | 0.47 |
| Clinical Events |  |  |  |  |  |  |
| Hospitalization | 1,362 | 1,194 | 167 | 146 | 11,440 | 0.87 |
| Symptom / Episode | 5,407 | 4,328 | 758 | 803 | 7,058 | 0.75 |
| Remission / Response | 1,035 | 703 | 323 | 273 | 11,648 | 0.68 |

Final Positive Cases After Consensus (n) denotes the number of positive instances in the adjudicated reference standard after resolution of annotation disagreements. Both Positive, Annotator A Only, Annotator B Only, and Both Negative summarize the initial independent annotations prior to consensus adjudication. Cohen’s κ was calculated using the initial independent annotations for each category. Higher κ values indicate stronger inter-rater agreement

### Supplementary Table S2. Impact of Fine-Tuning on Sentence-Level Clinical Event Extraction Performance

| Model | Symptom/ Episode F1 (no-FT) | Symptom/ Episode F1 (FT) | Hospitalization F1 (no-FT) | Hospitalization F1 (FT) | Remission/Response F1 (no-FT) | Remission/Response F1 (FT) |
| --- | --- | --- | --- | --- | --- | --- |
| LLaMA | 0.713 [0.703–0.722] | 0.880 [0.874–0.886] | 0.598 [0.578–0.616] | 0.920 [0.908–0.931] | 0.586 [0.561–0.610] | 0.790 [0.771–0.808] |
| MentaLLaMA | 0.561 [0.551–0.571] | 0.875 [0.869–0.882] | 0.142 [0.118–0.166] | 0.905 [0.894–0.916] | 0.024 [0.013–0.037] | 0.773 [0.753–0.794] |
| OpenBioLLM | 0.016 [0.011–0.021] | 0.872 [0.865–0.878] | 0.007 [0.002–0.014] | 0.920 [0.909–0.930] | 0.000 [0.000–0.000] | 0.779 [0.759–0.799] |
| Mistral | 0.403 [0.390–0.416] | 0.925 [0.920–0.930] | 0.512 [0.487–0.535] | 0.944 [0.934–0.952] | 0.170 [0.141–0.199] | 0.867 [0.851–0.883] |

Values are F1-scores with 95% confidence intervals. No-FT denotes the base model evaluated without task-specific fine-tuning. FT denotes the same model after LoRA fine-tuning on the annotated training dataset. Higher values indicate better performance.

### Supplementary Table S33. Precision and Recall for Chart-Level Temporal Feature Extraction

|  | Onset Precision | Onset Recall | Last Hospitalization Precision | Last Hospitalization Recall |
| --- | --- | --- | --- | --- |
| LLaMA_Ours | 0.560 | 0.549 | 0.628 | 0.631 |
| MentaLLaMA_Ours | 0.548 | 0.533 | 0.591 | 0.594 |
| OpenBioLLM_Ours | 0.553 | 0.539 | 0.617 | 0.620 |
| Mistral_Ours | 0.595 | 0.584 | 0.605 | 0.607 |

Precision and recall are reported for the two temporal chart-level prediction tasks: first episode onset time and most recent hospitalization. Values were calculated using exact-match evaluation after temporal normalization. Higher values indicate better performance.

### Supplementary Table S4. Oracle Analysis of Chart-Level Prediction Performance

| **Model** | **Method** | **Onset time (95%CI)** | **Episode count (95%CI)** | **Hospitalizations (95%CI)** | **Last hosp. (95%CI)** |
| --- | --- | --- | --- | --- | --- |
| LLaMA | Chain (Ours) | 0.744 [0.742–0.762] | 0.618 [0.400–0.910] | 0.670 [0.534–0.868] | 0.866 [0.546–1.000] |
|  | Oracle (gold Stage 1) | 0.728 [0.726–0.744] | 0.614 [0.396–0.904] | 0.672 [0.538–0.870] | 0.846 [0.528–1.000] |
| OpenBioLLM | Chain (Ours) | 0.738 [0.736–0.756] | 0.574 [0.354–0.868] | 0.682 [0.548–0.884] | 0.868 [0.534–1.000] |
|  | Oracle (gold Stage 1) | 0.776 [0.774–0.792] | 0.608 [0.390–0.900] | 0.668 [0.536–0.868] | 0.862 [0.530–1.000] |
| MentaLLaMA | Chain (Ours) | 0.734 [0.732–0.750] | 0.504 [0.282–0.806] | 0.610 [0.472–0.812] | 0.858 [0.536–1.000] |
|  | Oracle (gold Stage 1) | 0.762 [0.760–0.778] | 0.504 [0.268–0.816] | 0.614 [0.474–0.816] | 0.854 [0.526–1.000] |
| Mistral | Chain (Ours) | 0.772 [0.770–0.788] | 0.624 [0.406–0.912] | 0.676 [0.540–0.876] | 0.858 [0.540–1.000] |
|  | Oracle (gold Stage 1) | 0.702 [0.700–0.718] | 0.604 [0.380–0.908] | 0.656 [0.520–0.856] | 0.796 [0.480–1.000] |

Oracle analyses were performed by replacing Stage 1 model-generated sentence-level outputs with gold-standard sentence annotations. Chain (Ours) denotes the complete two-stage framework using model-generated sentence-level predictions. Oracle denotes chart-level prediction using gold-standard Stage 1 inputs. Values represent chart-level accuracy averaged across 10-fold cross-validation, with 95% confidence intervals shown in brackets.

### Supplementary Table S5. Prompt Sensitivity Analysis for Sentence-Level Information Extraction Using Non-Fine-Tuned LLaMA-3.1-8B-Instruct

| Variant | Description | Episode F1 | Hosp. F1 | Remission F1 | Age F1 | Time F1 | Persistence F1 | Vague F1 |
| --- | --- | --- | --- | --- | --- | --- | --- | --- |
| V1 – Baseline | Initial structured prompt | 0.511 [0.498–0.524] | 0.426 [0.402–0.451] | 0.363 [0.331–0.391] | 0.508 [0.456–0.556] | 0.506 [0.486–0.527] | 0.032 [0.008–0.065] | 0.137 [0.114–0.157] |
| V2 – No Examples | Prompt without few-shot examples | 0.751 [0.742–0.759] | 0.646 [0.626–0.666] | 0.526 [0.500–0.548] | 0.695 [0.658–0.729] | 0.684 [0.671–0.698] | 0.012 [0.000–0.030] | 0.078 [0.062–0.092] |
| V3 – Chain-of-Thought | Added step-by-step reasoning instructions | 0.738 [0.728–0.746] | 0.632 [0.612–0.652] | 0.526 [0.502–0.552] | 0.772 [0.739–0.805] | 0.784 [0.771–0.796] | 0.044 [0.018–0.081] | 0.189 [0.169–0.208] |
| V4 – Plain Text (Final) | Simplified plain-text output (selected) | 0.767 [0.759–0.775] | 0.643 [0.624–0.663] | 0.601 [0.573–0.625] | 0.742 [0.709–0.772] | 0.813 [0.801–0.825] | 0.074 [0.043–0.111] | 0.145 [0.117–0.175] |

Values are sentence-level F1-scores with 95% confidence intervals from prompt-only evaluations using non-fine-tuned LLaMA-3.1-8B-Instruct. V1 represents the initial structured prompt, V2 removes few-shot examples, V3 adds chain-of-thought reasoning instructions, and V4 uses the final simplified plain-text output format adopted in the proposed framework. Higher values indicate better performance.

### Stage 1. System Prompt of Sentence-level Extractor

Identify the type of medical events and corresponding temporal events from a given sentence in a clinical note.

**# Steps**

1. Analyze the Sentence: Break down the sentence to identify keywords or phrases that indicate a medical event type.

2. Determine Medical Event: Categorize the event as 'Episode/Symptom', 'Hospitalization', 'Remission/Response', or 'None' based on the context and identified keywords.

3. Identify Temporal Event: Recognize any temporal indicators to classify the temporal event as 'Age', 'Ago', 'Duration', 'Persistence', 'Date' or 'None'.

**# Output Format**

Provide the output as a JSON object with two fields, "medical event" and "temporal event", indicating the identified types.
{"medical event": "[Medical Event Type]", "temporal event": "[Temporal Event Type]"}

<demonstrations>

**Notes**
 - If the sentence does not clearly indicate any medical or temporal event, classify it as 'None' for one or both categories.
 - Pay close attention to temporal cues, as they are crucial for accurately identifying the time event type.
 - A sentence may contain multiple medical events; separate them with commas.

### Stage 2. System Prompt of Chart-level Extractor.

Please analyze each provided history of present illness summary and extract specific psychiatric information about the patient. Given triples (Setence, Medical_Event, Time_Event), follow these detailed instructions for each case summary:

1. **First Episode**: Determine the exact date or time period when the patient experienced their first onset of psychiatric disorder symptoms. This should include any initial diagnosis or significant symptoms. If no information is available, return "None."

2. **Number of Episodes**: Identify how many distinct episodes of the psychiatric disorder the patient has experienced. An "episode" is defined as the presence of psychiatric symptoms until there is partial or complete remission. Provide an exact number, ensuring a minimum of one if symptoms are mentioned.

3. **Number of Hospitalizations**: State the total number of times the patient was hospitalized due to their psychiatric disorder. Provide an exact count or return 0 if no hospitalizations are mentioned.

4. **Latest Hospitalization**: Specify the most recent date or period when the patient was hospitalized for psychiatric reasons. If not admitted to a hospital, return "None."

Please include all numbered headings and format the output in JSON.

\# Steps

- Read the summary carefully and note key details related to each of the four categories.

- If the supporting evidence or dates are conflicting or vague, choose the most relevant and recent information.

- Ensure clarity in extracting numbers and be specific about any estimations made.

\# Output Format

Output the results as a JSON object formatted as follows:

{

"first_episode": "[Time_Event or None]",

"number_of_episodes": "[Exact number]",

"number_of_hospitalizations": "[Exact number]",

"latest_hospitalization": "[Time_Event or None]"

}

\# Examples

<examples>

\# Notes

- Return "None" for First Episode and Latest Hospitalization only if clearly not available.

- For Number of Episodes and Number of Hospitalizations, more than "0" should be expected if symptoms are described.

- Use the fields in the given format to maintain consistency.

- In the case of multiple conflicting entries, prioritize the most recent or relevant information.

Remember that clarity and precision are key for each extracted element, maintaining ethical handling of the sensitive information provided in summaries.
